# How to Keep the Hypodermic Syringe in Order

**Published:** 1884-05

**Authors:** 


HOW TO KEEP THE HYPODERMIC SYRINGE IN ORDER.—Dr. William H. Morse, of New York, describes the following method of keeping the hypodermic syringe in order: "Unscrew the bowl of your syringe, take out the piston-rod, unscrew the *lower* piston *leather* and take it off, then put in its place a small piece of chamois-skin, and replace the leather and screw it tight. Now trim the chamois to the size of the bowl, and replace the piston, and the work is done. You will observe that you have only placed a bit of chamois *between* the *two* piston leathers. You now have a syringe which is in working order the *moment* water is introduced."—*Med. Record.*

